# Prognostic significance of CD103+ immune cells in solid tumor: a systemic review and meta-analysis

**DOI:** 10.1038/s41598-019-40527-4

**Published:** 2019-03-07

**Authors:** Younghoon Kim, Yunjoo Shin, Gyeong Hoon Kang

**Affiliations:** 1 Laboratory of Epigenetics, Cancer Research Institute, Seoul National University College of Medicine, Seoul, 03080 Korea; 2 Department of Pathology, Seoul National University College of Medicine, Seoul, 03080 Korea

## Abstract

CD103 is a transmembrane heterodimer complex that mediates cell adhesion, migration, and lymphocyte homing of cell through interaction with E-cadherin. Recently, CD103+ immune cells in human carcinoma has been investigated as a prognostic factor, however, the correlation between CD103+ immune cells and survival are still elusive. Therefore, a meta-analysis was performed to determine the prognostic value of CD103+ immune cells in solid tumor. Studies relevant to the subject was searched from PubMed, Embase, and Web of Science. Ten studies including 2,824 patients were eligible for the analysis. Tumors positive for CD103+ immune cells were associated with favorable overall survival, disease-free survival, and disease-specific survival. Subgroup analysis revealed that assessing CD103+ immune cells in epithelial and total (both epithelial and stromal) areas or using whole slide section were associated with good prognosis. Furthermore, stromal CD103+ immune cells or CD103+ immune cells evaluated by tissue microarrays were not always significantly prognostic. In conclusion, these results show that CD103+ immune cells are associated with prognosis in solid tumor. However, the region of assessment and selection of material for the evaluation could affect the value of CD103 as a prognostic biomarker.

## Introduction

CD103, also known as integrin αEβ7 (ITGAE), is a transmembrane heterodimer complex that is involved in cell to cell or cell to matrix interaction^1^. CD103 mediates cell adhesion, migration, and lymphocyte homing of cell through interaction with E-cadherin, which is expressed in epithelial cells^2^. Therefore, CD103 is considered a hall mark of specific subset of immune cells that resides within the epithelium of mucosal organs including genital tract, stomach, lung, and skin^3–6^. The immune cells that express CD103 includes effector memory CD8+ T cells, regulatory CD8+ T cells (Tregs), and CD11c-high/MHC class II-high dendritic cells^6–9^.

In recent years, the importance of CD103+ immune cells in cancer has grown due to its intimate relationship with immune checkpoint and *BRAF* blockade. In ovarian cancer, intraepithelial CD8+ tumor-infiltrating lymphocytes (TILs) that expressed CD103 were suspected to cause exhausted phenotype by chronic stimulation that leads to resistance in immune therapy, and CD103 was widely co-expressed with PD-1^10^. In rodent melanoma, expansion and activation of CD103+ dendritic cells were the only antigen presenting cells that transported intact antigen which led to enhanced response of PD-L1 and *BRAF* target therapies^11^. CD103+ immune cells were also a predictive marker of immunotherapy in lung cancer and increased expression was observed following anti-PD-1 therapy in ovarian cancer and melanoma^12–14^.

A number of studies suggests that CD103+ TIL in human malignancy is associated with prognosis^15–18^. However, this characteristic of CD103+ immune cells in cancer are still elusive with the exception of a meta-analysis of ovarian cancer^19^. Some studies define CD103 as predictive markers of intraepithelial CD8+ T cells or Tregs^20,21^, but other studies show that CD103+ immune cells are present within the stroma^22^ and could be considered to be associated with survival^23^. Moreover, recent studies have shown that the usage of tissue microarray (TMA) instead of whole tissue section slides could have issues when evaluating TIL within tumors^24,25^ but a comparison between TMA and whole section has not been addressed for CD103+ immune cells.

In this study, we conducted a meta-analysis to determine the prognostic significance of CD103+ immune cells in solid tumors. The aim of this study is to clarify the prognostic role of CD103 expression in immune cell as a biomarker across multiple tumors and to verify which region within tumor (epithelial, stromal, or both) and material (TMA or whole section) should be used in the assessment of the marker.

## Results

### Eligible studies and study characteristics

**Figure 1:**
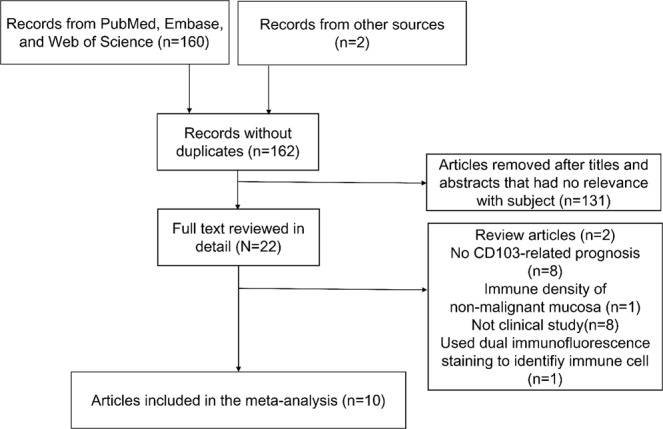
Flow chart of the selection process for meta-analysis.

A total of 160 records were found from PubMed, Embase, and Web of Science after removing repeated records. Two records were added from references of a previous meta-analysis^19^. After the titles and abstracts were reviewed, 131 records were removed due to the irrelevance with prognosis. Full text of remaining 31 records were reviewed in detail. Among the remaining records, 3 were reviews, 9 had no CD103-related prognostic value, 8 were not clinical studies, and one used dual immunofluorescence staining to identify CD103+ CD8+ T cells. In the end, 10 articles with 2,824 patients were selected as eligible for the meta-analysis (Fig. 1). Most studies dichotomized the patients in to high (positive) and low (negative), but a single study^22^ used continuous value for evaluation. The median sample size of all studies was 257.5, with a close range from 98 to 485. Seven of the studies were tumors from the genitourinary tract, followed by two studies from lung, and a single study from breast (Table 1).

**Table 1:** Characteristics of articles included in the meta-analysis.

Author	Year	Cancer	Material	Cut-off value	Region	Endpoint	Patient	CD103+ (%)
Djenidi^22^	2015	Non-small cell lung cancer	WS	none (continuous)	all three	OS, DFS	98	—
Wang^16^	2016	Breast cancer	TMA	90th percentile	epithelium	OS	424	43 (10.1)
Wang^33^	2015	Urothelial cell carcinoma	WS	>9cells/HPF	epithelium	OS	262	148 (56.5)
Bosmuller^34^	2016	HGSC	TMA	>1 cell/HPF	epithelium	OS	202	140 (69.3)
Santoiemma^26^	2016	Ovarian cancer	TMA	≥10 cells/core	epithelium	OS	135	31 (23.0)
Koh^23^	2017	Squamous cell lung cancer	TMA	mean	epithelium/stroma	OS, DFS	378	108/111 (28.6/29.4)^a^
Workel^20^	2016	Endometrial adenocarcinoma	TMA	>27.28 cells/mm^2^	total	DFS, DSS	253	84 (33.2)
Komdeur^35^	2017	Cervical cancer	TMA	>29 cells/mm^2^	total	DFS, DSS	387	200 (51.7)
Zhou^27^	2018	Renal cell carcinoma	WS	>1 cell/HPF	total	DSS	200	117 (58.5)
Webb^36^	2013	Ovarian cancer	TMA	≥5(1) cells/core^b^	TMA	DSS	485	339 (69.9)

^*^OS: overall survival, DFS: disese-free survival, DSS: disease-specific survival, WS: whole slide, TMA: tissue microarray, HPF: high power field, HGSC: high grade serous ovarian cancer.

^a^Positive for epithelial area/positive for stromal area.

^b^≥5 cells/core to evaluate prognosis, ≥1 cell/core to evaluate positivity.

### Meta-analysis

**Figure 2:**
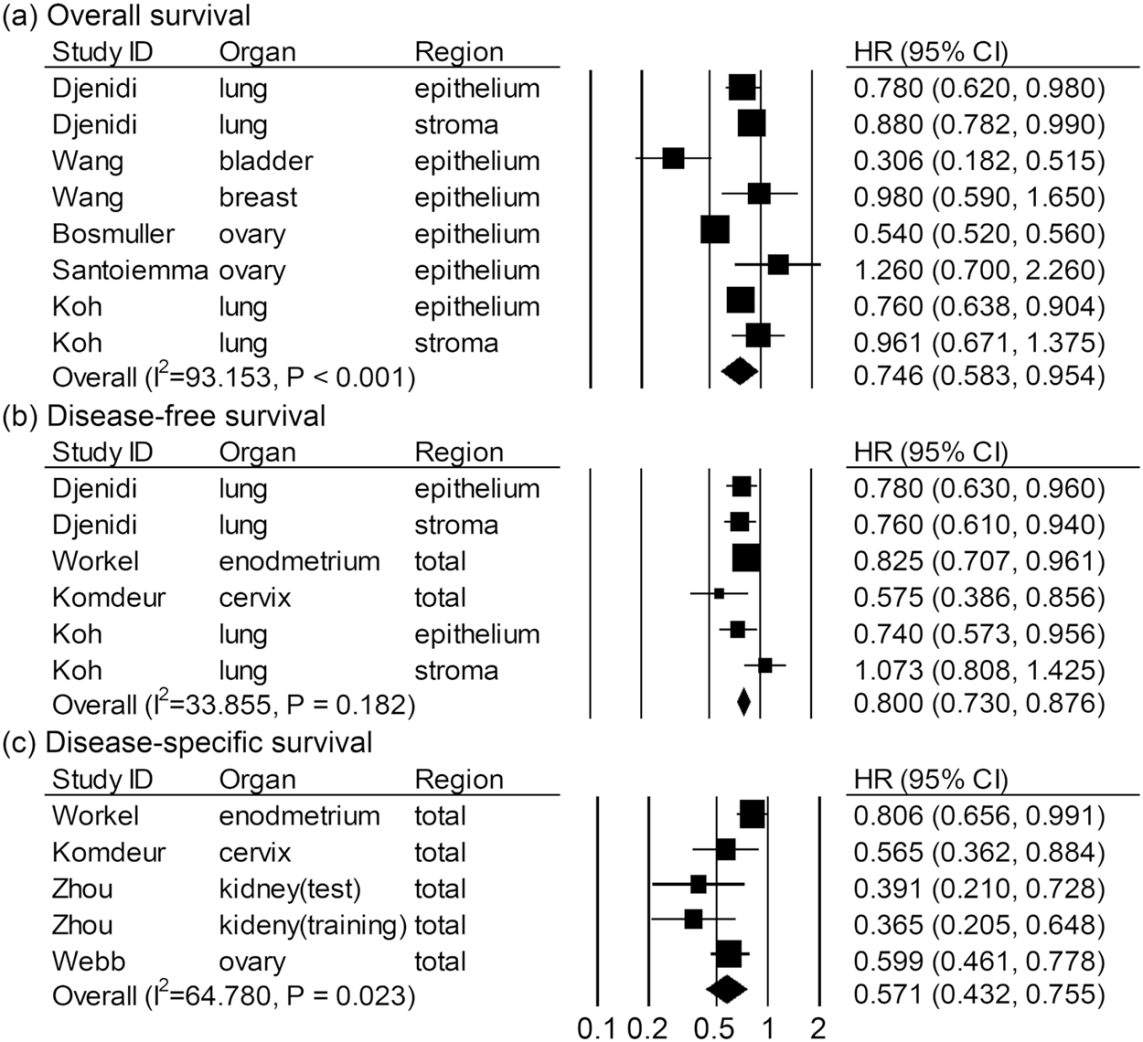
Correlation between CD103+ immune cells and survival in solid tumors. (**a**) Overall survival. (**b**) Disease-free survival. (**c**) Disease-specific survival.

The meta-analysis between CD103+ immune cells and prognostic value of overall survival (OS) involved six articles with eight effect sizes (ES) (Fig. 2a). CD103+ immune cells were associated with favorable OS in solid tumor (HR = 0.746, 95% CI = 0.583–0.954, *P* value = 0.020). A random effect model was applied since heterogeneity test showed *P* value < 0.001 and *I*^2^ = 93.153. Fours studies with six ES included the correlation between CD103+ immune cells and disease-free survival (DFS) (Fig. 2b). In a fixed effect model (*P* value = 0.182 and *I*^2^ = 33.855), CD103+ immune cells were associated with prolonged DFS in solid tumor (HR = 0.800, 95% CI = 0.730–0.876, *P* value < 0.001). Survival outcomes involving smaller number of articles, disease-specific survival (DSS) (n = 4) had better prognosis in patients with CD103+ immune cells than those with CD103- immune cells in random effect model (HR = 0.571, 95% CI = 0.432–0.755, *P* value < 0.001) (Fig. 2c). In a subgroup analysis (Table 2), studies evaluating the immune cells of stromal area were showed significant correlation between CD103+ immune cells and OS (HR = 0.888, 95% CI = 0.794–0.993, *P* value = 0.037) but not DFS (HR = 0.892, 95% CI = 0.636–1.250, *P* value = 0.506). Studies using TMA had a significant correlation between CD103+ immune cells and DFS (HR = 0.805, 95% CI = 0.661–0.982, *P* value = 0.032) but not between CD103+ immune cells and OS (HR = 0.809, 95% CI = 0.595–1.100, *P* value = 0.176). However, studies using whole section or those that evaluate CD103+ immune cells of the epithelial or the total area always maintained correlation with survival regardless of the type of outcome.

**Table 2:** Subgroup analysis of included studies.

Outcome	Subgroup	Effect Model	N of studies	HR	95%CI LL	95%CI UL	*P* value
OS	Epithelium	Random	6	0.688	0.528	0.896	0.006*
OS	Stroma	Fixed	2	0.888	0.794	0.993	0.037*
OS	TMA	Random	4	0.809	0.595	1.100	0.176
OS	WS	Random	2	0.654	0.443	0.968	0.034*
OS	Lung(epithelium)	Fixed	2	0.767	0.668	0.881	<0.001**
OS	Ovary	Random	2	0.783	0.343	1.784	0.560
DFS	Epithelium	Fixed	2	0.764	0.649	0.898	0.001*
DFS	Stroma	Random	2	0.892	0.636	1.250	0.506
DFS	Total	Random	3	0.802	0.701	0.918	0.001*
DFS	TMA	Random	3	0.805	0.661	0.982	0.032*
DSS	TMA	Random	3	0.571	0.432	0.755	<0.001*

^*^P value < 0.05, **P value < 0.001.

### Publication bias and sensitivity analysis

**Figure 3:**
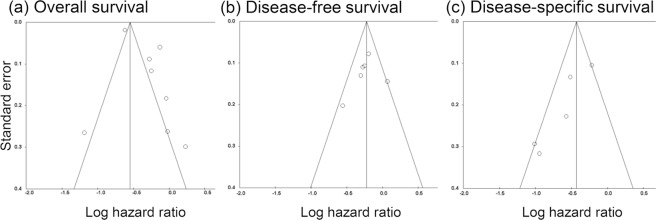
Funnel plot for studies included in the meta-analysis. (**a**) Overall survival. (**b**) Disease-free survival. (**c**) Disease-specific survival.

**Figure 4:**
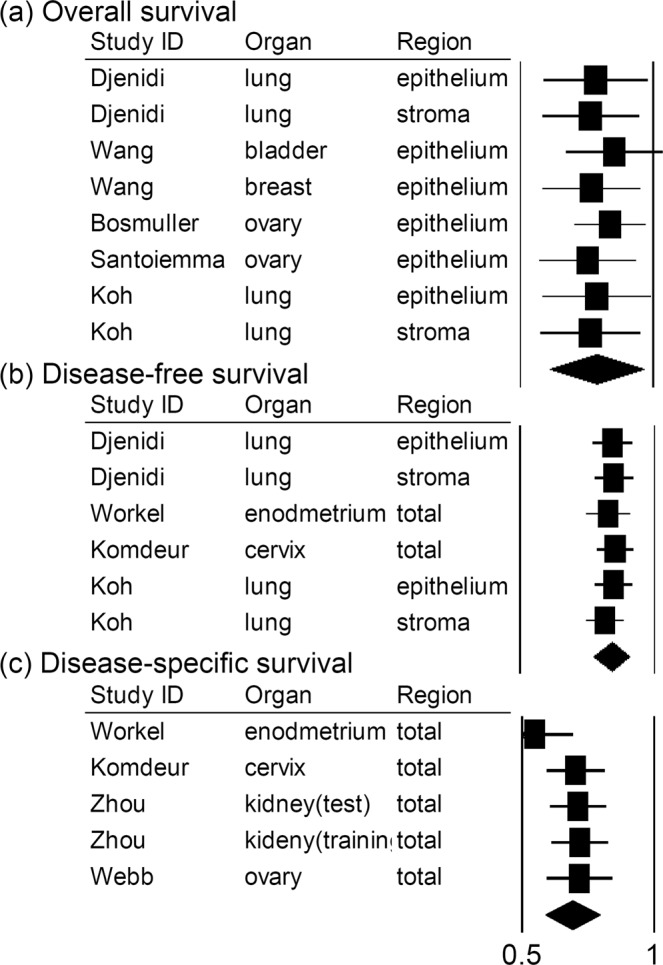
Sensitivity analysis of the meta-analysis. (**a**) Overall survival. (**b**) Disease-free survival. (**c**) Disease-specific survival.

Begg’s funnel plot and Egg’s test were used to evaluate potential publication bias (Fig. 3). According to Egg’s test, OS and DFS did not show publication bias (*P* value = 0.090 and *P* value = 0.600, respectively) but DSS was significant for publication bias (*P* value = 0.029). Removing a single study from the meta-analysis did not have significant effect on the overall conclusion apart from a single study from Wang *et al*. that involved urothelial cell carcinoma (Fig. 4).

## Discussion

Expression of CD103 has been suggested as a marker of prolonged survival in solid tumors as well as a predictive marker of immunotherapy in recent years. However, the expression of CD103 has been reported in immune cells^6–9^ which limits the comprehensiveness of the subject of individual articles since most studies focus on a single material (TMA or whole slide) and a single region of interest (intraepithelial, stromal, or total areas) when evaluating CD103+ immune cells. In this meta-analysis, for the first time, the correlation between CD103+ immune cells and prognosis has been explored via pooled analysis of solid tumors.

A total of 2,824 patient from 10 different studies was included in the meta-analysis. The results showed that OS (HR = 0.746, 95% CI = 0.583–0.954, *P* value < 0.020), DFS (HR = 0.800, 95% CI = 0.730–0.876, *P* value < 0.001), and DSS (HR = 0.571, 95% CI = 0.432–0.755, *P* value < 0.001) are all indicative measurements of the prognostic value of CD103+ immune cells in solid tumors. Subgroup analysis revealed that CD103+ immune cells were not significantly correlated with OS when TMA studies were pooled and stromal CD103+ immune cells were not prognostic markers of DFS. On the contrary, pooled studies for whole slide or studies that investigated on intraepithelial or total CD103+ immune cells were determined prognostic whenever subgrouping was available.

The meta-analysis unveils novel characteristics of CD103+ immune cells within solid tumors. Although expression of CD103 was also observed in the stromal area, prognostic impact was only significant when CD103+ immune cells of epithelial or those of both epithelial and stromal (total) areas were accounted for. A pooled survival analysis of studies using TMA where not significantly correlated with OS but still maintained prognostic for DFS and DSS. This might be considered as a short side for TMA, but when reviewing the counting strategies of each article, studies using TMA counted multiple cores with 0.6 mm diameter or a single core with 2.0 mm diameter^16,23,26^, whereas studies that used whole section counted not the entire slide but 3 to 5 microscopic fields within a given slide^22,27^. Moreover, studies that used TMA had a larger average number of patients compared with that of studies with whole section (323.4 vs 186.7). Another caveat is the fact that not enough studies were available to compare the effect of TMA and whole section according to each tumor. Therefore, for better prognostic resolution of CD103, assessing intraepithelial or total CD103+ immune cells and using multiple TMA cores per sample with large diameter could be suggested in a large scale study.

Human malignancy and its microenvironment shows diversity according to genetic background and etiology, and a pooled analysis could undermine unique characteristics of each tumor type. In our current study, subgroup analysis according to tumor type was available in two out of seven different types included in the meta-analysis, and not all tumor types were significantly associated with prognosis. Therefore, although the current study provides meaningful findings in survival-related features of CD103+ immune cells across solid tumors and various materials used for assessment, it should be noted that the correlation between CD103+ immune cells and prognosis could vary among different tumor types. Further investigation is required to fully elucidate clinical value of CD103+ immune cells in prognostic predictions.

Other limitations of this study are as follows. In our study, the subtype of CD103+ immune cells that correlates with prognosis is still poorly determined because none of the included studies used double staining immunohistochemistry or multiplex immunohistochemistry to identify if the immune cells are TIL although most studies defined CD103+ immune cells as CD103+ TIL. Furthermore, relatively small number of studies were available for the pooled analysis, which lead to exclusion of solid tumors of major systems, such as gastrointestinal tract. Since colorectal cancer has shown different prognostic meaning for FOXP3+ Treg compared with other organs in previous studies^28–30^, and CD103+ immune cells are widely expressed in Tregs, the inclusion of some organs has the potential to shift the result. Finally, Egg’s test determined that the result between CD103+ immune cells and DSS has a publication bias.

In conclusion, CD103+ immune cells are markers of favorable prognosis when effect sizes for survival analysis of solid tumors from various organs where pooled and meta-analyzed, although prognostic feature between tumor types may vary. For further research, to find out the full spectrum of prognostic value and therapeutic potential of CD103, we suggest that immune cell subtyping, optimal tumor region selection, and proper material selection for evaluating CD103+ immune cells that could suffice in producing data for large sample size are necessary.

## Materials and Methods

### Publication search strategy

Articles relevant with the subject were retrieved from electronic databases, PubMed, Embase, and Web of Science until June 1^st^ of 2018. The search term were ‘CD103’, ‘cancer, carcinoma or tumor’, and ‘prognosis, prognostic or survival’. Additional articles were retrieved from reference cited in the eligible studies and a meta-analysis article of the ovarian cancer^19^. The results were limited to English articles based on human malignancies. Each source and collecting period of tumor samples were carefully recorded to avoid studies with identical patient population. When duplicate data was found, the most recent iteration of the cohort was included.

### Inclusion and exclusion criteria

Studies eligible for the inclusion were: (1) studies that measured expression of CD103 by immunohistochemical (IHC) staining in immune cells within any human tumor samples, (2) studies that report the correlation between CD103 expression within the primary tumor and survival, (3) studies that offer hazard ratio (HR) and 95% confidence intervals (CI) directly from the main article or supplementary materials or provide alternative values that could estimate HR and 95% CIs. Exclusion criteria of this study were: (1) studies that were from abstracts of conferences, reviews, and comments, (2) studies that were based on xenograft models or human hematopoietic or lymphoid malignancies, (3) studies that had insufficient pathological or survival data which led to unavailability to extract HR and 95% CIs.

### Data extraction and quality assessment

Two reviewers (YK and GK) independently collected data from eligible articles according to the criteria. Newcastle-Ottawa Scale (NOS) was used to evaluate quality of all studies. Scores equal to or higher than 6 out of 9 was defined as qualified. Parameters that were extracted from each study were as follows: surname of first author, year of publication, primary organ and type of cancer, material and regions of assessment for CD103, cut-off value for CD103+ immune cells, type of outcome, and number of analyzed patients. Meta-analysis of CD103+ immune cells were measured by four different outcome endpoints including OS, DFS, and DSS. Survival data were extracted as HR and 95% CIs from Cox univariate analysis. For articles with only Kaplan-Meiere curves, HR and 95% CIs were estimated by using Engauge Digitizer V9.8 (http://digitizer.sourceforge.net) and spreadsheets provided by Tierney *et al*.^31^ and Altman *et al*.^32^. However, this estimate was only available when number of patients from the research arm and control arm were reported. For this reason, we contacted the authors of articles that missed on this information, which one of the authors responded and provided patient numbers for each arm^23^.

### Statistical analysis

The correlation between CD103+ immune cells and prognosis of patients with solid tumor were measured via meta-analysis. Pooled HR and 95% CIs for OS, DFS, and DSS were calculated by using Comprehensive meta-analysis V3.3 (Biostat, Englewood, NJ, USA). Cochran’s *Q* and *I*^2^ were used to determine statistical heterogeneity. Subgroup analysis was performed when multiple studies were in each subgroup. *P* value <0.05 or *I*^2^> 50 were considered to have presence of heterogeneity and random effect model was used instead of fixed effect model. Sensitivity analysis was conducted to find out the effect of a single study to the pooled analysis. A graphical funnel plot and Egg’s test were used to evaluate potential publication bias. All tests were two-sided and *P* value < 0.05 was considered as statistically significant, except for tests for heterogeneity.

## Data Availability

All Relevant data are within the paper.
